# Single-cell transcriptomic analyses of T cells in chronic HCV-infected patients dominated by DAA-induced interferon signaling changes

**DOI:** 10.1371/journal.ppat.1009799

**Published:** 2021-08-09

**Authors:** Matthew A. Burchill, Matthew P. Salomon, Lucy Golden-Mason, Amanda Wieland, Ana C. Maretti-Mira, Michael Gale, Hugo R. Rosen

**Affiliations:** 1 Division of Gastroenterology & Hepatology, University of Colorado, Aurora, Colorado, United States of America; 2 Department of Medicine, Keck School of Medicine, University of Southern California, Los Angeles, California, United States of America; 3 Division of Gastrointestinal and Liver Diseases, Keck School of Medicine, University of Southern California, Los Angeles, California, United States of America; 4 Research Center for Liver Disease (RCLD), University of Southern California, Los Angeles, California, United States of America; 5 Center for Innate Immunity and Immune Disease, Department of Immunology, University of Washington, Seattle, Washington, United States of America; University of Alabama at Birmingham, UNITED STATES

## Abstract

Chronic infection with HCV is manifested by dysregulation of innate immune responses and impaired T cell function at multiple levels. These changes may impact susceptibility to other infections, responsiveness to antiviral therapies, vaccine responsiveness, and development of complications such as hepatocellular carcinoma. Highly effective direct-acting antiviral (DAA) therapy has revolutionized the management of chronic HCV, with expected cure rates exceeding 95%. DAA treatment represents a unique opportunity to investigate to what extent elimination of viral replication and chronic antigen stimulation can restore immunologic phenotype. In this study we interrogated the global transcriptional profile of isolated peripheral blood T cells before, during and after IFN-free DAA therapy using single-cell mRNA sequencing. Our results demonstrate that T cells mapped at single-cell resolution have dramatic transcriptomic changes early after initiation of DAA and many of these changes are sustained after completion of DAA therapy. Specifically, we see a significant reduction in transcripts associated with innate immune activation and interferon signaling such as *ISG15*, *ISG20*, *IFIT3*, *OAS* and *MX1* in many different T cell subsets. Furthermore, we find an early upregulation of a gene involved in suppression of immune activation, *DUSP1*, in circulating T cells. *Conclusion*: This study provides the first in-depth transcriptomic analysis at the single-cell level of patients undergoing DAA therapy, demonstrating that IFN-free antiviral therapy in chronic HCV infection induces hitherto unrecognized shifts in innate immune and interferon signaling within T cell populations early, during, and long-term after treatment. The present study provides a rich data source to explore the effects of DAA treatment on bulk T cells.

## Author summary

Direct-acting antiviral (DAA) therapy can now cure hepatitis C viral (HCV) infection in greater than 95% of individuals. Chronic infection with HCV causes changes in immune T cells which may leave individuals more susceptible to other infections even after successful therapy. We used a revolutionary technology which allows us to examine gene expression in individual T cells before, during and after DAA treatment. We identified 15 distinct circulating T cell groups. We find significant changes in gene expression early after initiation of treatment many of which are sustained at twelve weeks after successful completion of treatment. There is downregulation of innate immune activation in several T cell subsets. Our study provides a rich data source to explore the effects of DAA treatment on bulk T cells.

## Introduction

Hepatitis C virus (HCV) is the world’s most common blood-borne viral infection for which there is no vaccine [1]. Viral persistence is established in the majority of infected subjects, indicating HCV successfully subverts innate and adaptive immune responses at multiple levels. Chronic HCV is a leading cause of hepatitis, cirrhosis, liver cancer, and indication for liver transplantation in the Western world [2]. Moreover, we and others have demonstrated that chronic HCV infection has detrimental effects on global innate and adaptive immune responses [3–9]. These alterations in global immunity result in an increased incidence of respiratory infections [10], variable HBV vaccine responses [11] and alterations in the microbiota in individuals with chronic HCV [12]. Highly effective direct-acting antiviral (DAA) therapy has revolutionized the management of chronic HCV [13], with standard cure rates exceeding 95% [14]. Multiple groups have shown that following IFN-free DAA therapy, there may be partial restoration of both innate and adaptive immune homeostasis [3,15–18].

T cells are the primary effector cells that mediate viral clearance in spontaneous recovery from HCV infection through the secretion of antiviral cytokines and cytolytic activity [19]. In order to comprehensively examine the role of DAA-mediated HCV cure on global T cell immune homeostasis, we utilized single-cell sequencing to characterize the transcriptome of circulating T cells prior to, during, and after DAA-mediated HCV cure. Single-cell RNA-seq identified 15 distinct T cell clusters that were tracked over time. Using enrichment analyses of signaling pathways, we demonstrated a dramatic reduction in the signaling pathway downstream of type 1 interferon (IFN) in a majority of T cells in the peripheral blood. Particular subsets of chronically activated T cells have a much greater magnitude of transcriptional re-setting following DAA therapy. We also found a transient increase in expression of *DUSP1* (implicated in negatively regulating pro-inflammatory cytokine production by innate immune cells) and AP-1 family members in a majority of T cells at treatment week 4, reaching levels detected in normal control subjects, suggesting normalization of T cells early that is not sustained post treatment. Together, these data identify rapidly-normalized IFN signaling as a predominant response to DAA-mediated cure but point to persistent defects that might represent potential targets to alleviate sequelae of the global immune dysregulation observed during chronic viral infections.

## Results

### Single-cell transcriptome profiling of T cells in chronic HCV infection

In order to examine the nature of CD4^+^ and CD8^+^ T cell responses, scRNA-seq transcriptomic analyses were performed at baseline, 4 weeks into treatment, and 12 weeks post-treatment completion. Importantly, DAA therapy led to a rapid decline of viremia and a sustained virological response (SVR), defined as undetectable serum HCV RNA 12 weeks post-treatment in all patients (**S1 Table**). Specifically, we profiled CD3^+^ T cells by using droplet-based single-cell RNA sequencing technology (10X Genomics) from 6 men (aged 43 to 67 years) with chronic HCV genotype 1 infection before, during and after treatment with ledipasvir/sofosbuvir (**S1 Table**). Using this strategy, the total number of recovered T cells was 63,183 comprising 19,354 cells for pre-treatment (mean: 3,226 cells/patient), 19,461 for 4 weeks into treatment (mean: 3,244 cells/patient), and 24,368 for 12 weeks post-treatment (mean: 4,061 cells/patient). We also included a cohort of normal control subjects for comparison.

**Fig 1:**
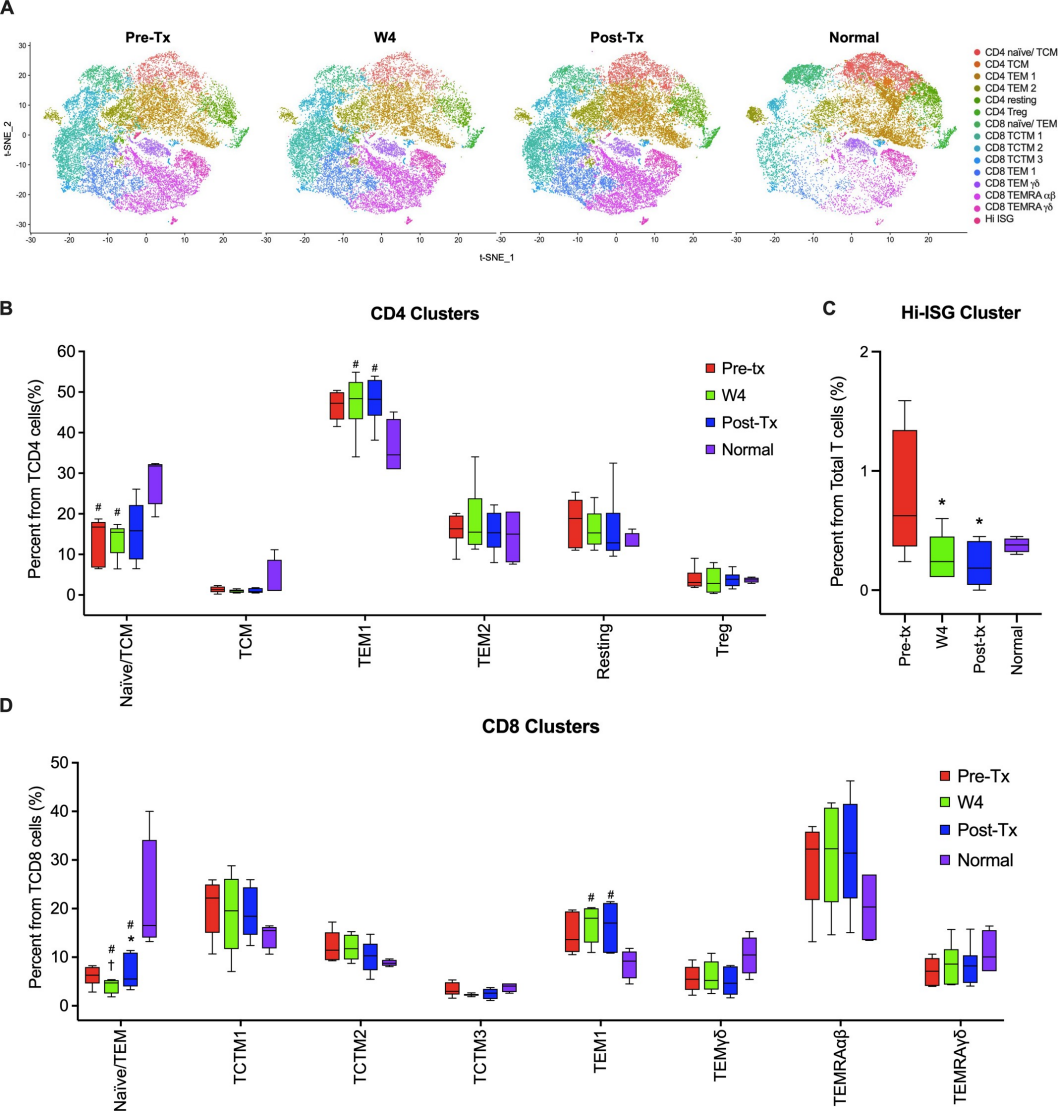
Overall distribution of peripheral blood T cell populations. (**A**) Clustering revealed 15 distinct T cell populations represented here in two-dimensional space using t-Distributed Stochastic Neighbor Emending (tSNE). All cells from each patient (n = 6) and time points are plotted, including cells from normal healthy controls (n = 3). Global changes in T cell subpopulations frequencies were analyzed during DAA therapy and compared to healthy controls. (**B**) The frequencies of the six CD4 clusters during and after DAA therapy and those found in normal control subjects is shown. (**C**) The frequency of the Hi-ISG cluster decreased to normal levels during and after therapy in HCV patients when compared to the samples before starting DAA treatment. (**D**) The frequencies of the eight CD8 clusters during and after DAA therapy and those found in normal control subjects is shown. Differences among time points within a cluster were tested using Friedman’s One-Way ANOVA for the HCV paired samples, and Kruskal-Wallis to compare HCV samples to healthy controls. *Significant compared to Pre-Tx; ^†^Significant compared to Post-Tx; ^#^Significant compared to Normal. TCM: T central memory; TEM: T effector memory; Treg: T regulatory; TCTM: T central-transitional memory; TEMRA: Terminally Differentiated Effector Memory; Tx: DAA Treatment.

Based on their expression profiles, we visualized the cells in 2D space using t-distributed stochastic neighbor embedding (tSNE) [20], an established method for nonlinear dimensionality reduction (**Fig 1A**). Differential gene expression from the scRNA-seq data resolved T cell subsets [21] and putative functional states into 15 independent clusters (all expressing *CD3ε* and a T cell receptor constant region). The relative gene expression of *CD4* and *CD8A* and classical marker genes was also examined to assign groups to different lineages and effector phenotypes.

CD4^+^ T cells comprised 6 clusters: 2 groups of CD4 naïve/central memory T (TCM) cells (relatively higher expression of *CCR7*, *CD27*, *SELL*, and *TCF7)*, 2 distinct populations of CD4 effector memory cells (TEM), CD4 resting, and CD4 regulatory T cells (Treg). CD8^+^ T cells comprised 8 clusters distinct from CD4^+^ T cells including a naïve/effector memory population, 3 central/transitional memory populations (TCTM) two clusters representing effector memory, 2 clusters representing terminally differentiated effector cells (TEMRA) that express high levels of cytotoxic markers *PRF1* and *NKG7*; [22,23], one of the TEMRA clusters expressed the traditional α/β chains and the other γ/δ chains. In addition, we identified a group of cells that included both CD4 and CD8 expressing T cells that were clustered by high expression levels of interferon-stimulated genes (ISGs,Hi-ISG cluster). The relative distribution of the peripheral T cell populations during treatment and compared to normal control subjects is shown in **Fig 1B–1D**. The frequency of the CD4 clusters does not significantly change with DAA treatment, however, compared to normal controls, pre- and W4 of treatment Naïve/TCMs are decreased and the TEM1 cluster is increased at week 4 and post-treatment. A similar pattern is seen for CD8 clusters with the CD8 Naïve/TEM population decreased at W4 and post-treatment and the CD8 TEM1 increased compared to control subjects. The CD8 naïve/TEM population does increase compared to pre-treatment, however, does not reach levels seen in normal controls. The Hi-ISG cluster decreases at W4 of treatment and remains at levels seen in controls post-treatment.

**Fig 2:**
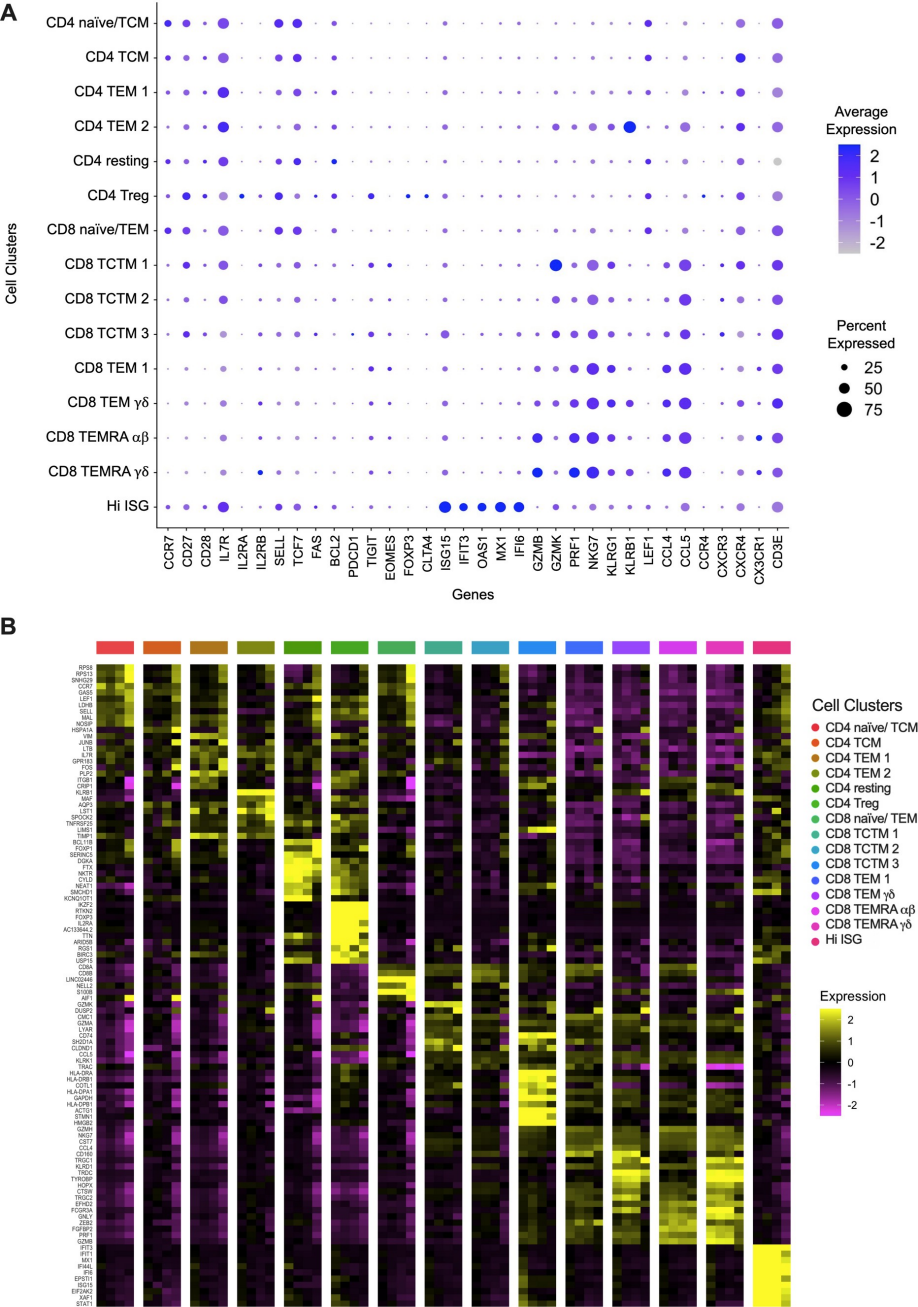
Expression of marker genes among T cell populations. (**A**) The dotplot shows the canonical molecular markers expressed by the T cell population clusters and that were used to classify the different T cell subpopulations. (**B**) Heatmap displaying the hierarchical clustering of the 10 most variable genes among the detected clusters. Only differentially expressed genes are shown. TCM: T central memory; TEM: T effector memory; Treg: T regulatory; TCTM: T central-transitional memory; TEMRA: Terminally Differentiated Effector Memory.

**Fig 2A** demonstrates the relative expression of canonical genes demarcating “classic” naïve, effector, memory and regulatory populations [23,24] according to the clusters generated through our analyses. The clusters in each individual patient are demonstrated in **S1 Fig**. **Fig 2B** demonstrates the heatmap of ten of the most differentially regulated genes for each T cell cluster. This high-resolution and unbiased detection of differences confirmed the assignment of cell clusters by correlation with putative function (**Fig 2A**) and expanded direct comparison of gene expression between similar subsets. As expected, the CD4T reg cluster expresses high levels of *FOXP3*, *IL2RA* and *CTLA4* [21]. Recently, the expression of TNF family molecule lymphotoxin β (*LTB)* was found to be elevated in CD4^+^ T cells that were precursors for cytotoxic CD4^+^ T cells [25]. Similary, we found *LTB* expression to be very high in CD4 effector memory T cell cluster 1 (TEM1) suggesting that this population is a precursor to a CTL- like CD4^+^ T cell. Additionally, we find an unique cluster of CD4 effector memory T (TEM2) cells that are defined by their high expression of *KLRB1*. Previous studies have demonstrated that this subset marks CD4^+^ cells that produce high levels of the cytokines IFNγ, TNFα and IL17 [26,27]. Finally, we observe a unique population of T cells containing both CD8^+^ and CD4^+^ T cells that demonstrated the highest expression of interferon-stimulated genes (*IFIT3*, *IFIT1*, *MX1*, *IFI44L*, *IFI6*, *ISG15* and *STAT1*) (**Fig 2B**) which we named “Hi-ISG”.

### DAA-mediated cure results in sustained changes in innate immune and cytokine signaling pathways within many T cell clusters

**Fig 3:**
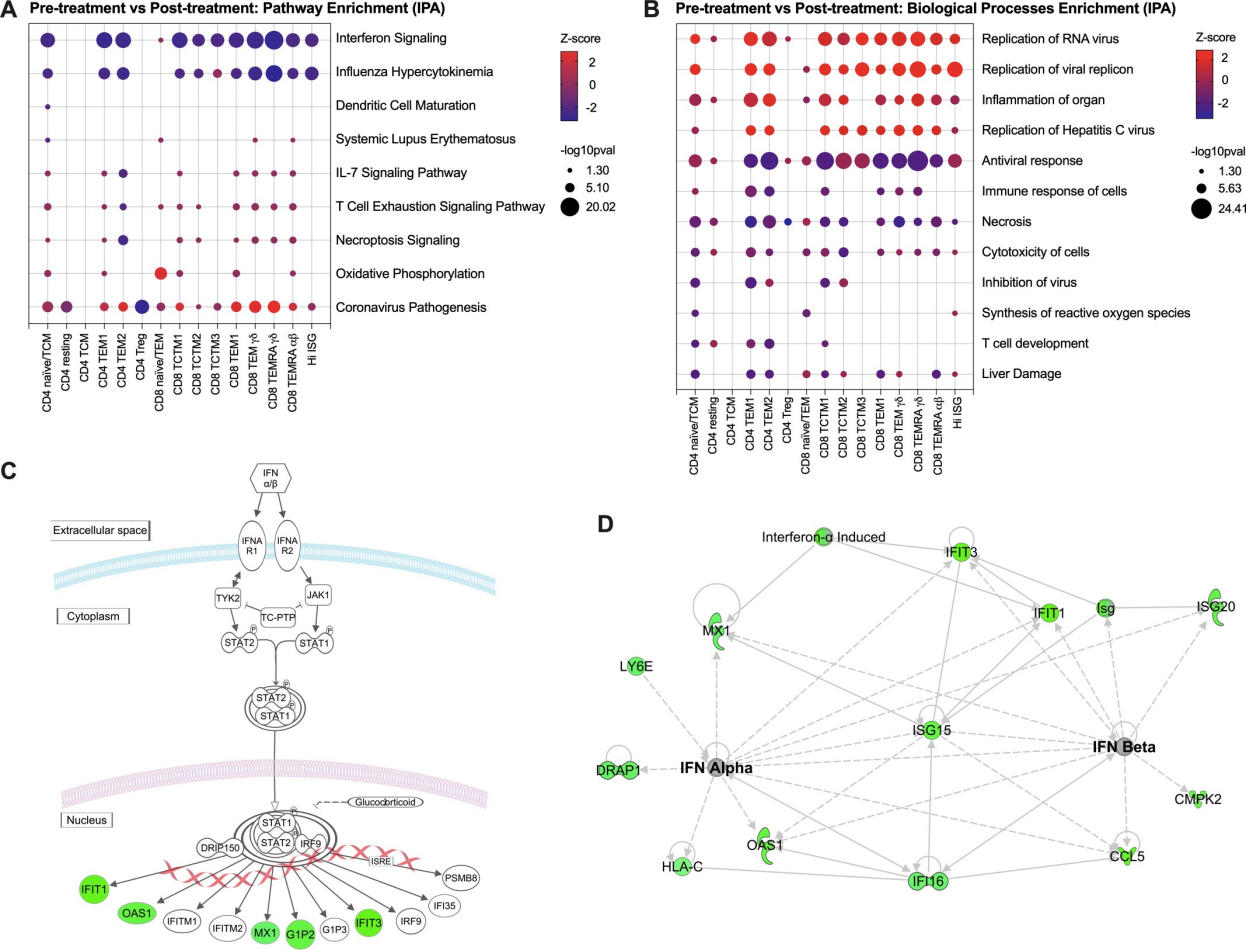
Enrichment analysis of differentially expressed genes between post and pre-treatment for each T cell cluster. (**A**) Plot of the top significant enriched IPA pathways among T cell clusters, for significantly up- and downregulated genes, showing z-score and -log10pvalues. (**B**) Plot showing the comparison of top significantly enriched biological functions (IPA) among T cell clusters, z-score and -log10pvalues indicated direction and significance, respectively. (**C**) Cartoon representation of the IFN signaling in the Hi-ISG T cell cluster. Genes in green are significantly downregulated comparing post-treatment to pre-treatment samples. (**D**) Gene-concept network between interferon alpha/beta signaling for the set of significantly differentially expressed genes for the Hi-ISG T cell cluster. Genes in green are significantly downregulated comparing post-treatment to pre-treatment. TCM: T central memory; TEM: T effector memory; Treg: T regulatory; TCTM: T central-transitional memory; TEMRA: Terminally Differentiated Effector Memory.

In order to identify signaling pathways involved in changes within T cells induced by DAA-mediated HCV cure, we performed differential expression analysis within the different clusters prior to and after DAA-mediated cure. **Fig 3A** shows the top significant enriched IPA pathways among T cell clusters associated with HCV cure in peripheral blood T cells. As evident from this analysis, we find signifcant changes in genes associated with innate immune activation and interferon signaling in viral infection. Furthermore using IPA to examine biological processes enrichment (**Fig 3B**), we find many processes that are enriched and shared across the different T cells clusters when comparing 12 weeks after completion of DAA therapy to pre-treatment time points. The CD4 central memory and effector cell populations, CD8 effector memory and TEMRA populations, and Hi-ISG populations demonstrate the most significant transcriptional changes with HCV cure. DAA therapy results in a change in many canonical mediators of innate immune and interferon antiviral response including *IFIT1/3*, *OAS1* and *MX1***. Fig 3C** depicts the observed down-regulation of interferon signaling and **Fig 3D** shows the gene-concept network between interferon alpha/beta signaling for the set of significantly differentially expressed genes for the Hi-ISG T cell cluster when comparing post-treatment to pre-treatment samples. Together, this unbiased dataset enrichment demonstrates that treatment with DAA therapy results in the rapid down-regulation of systemic innate immune activation and IFN reponse in T cells.

### Rapid kinetics of DAA treatment effects on the transcriptional profile of T cells

**Fig 4:**
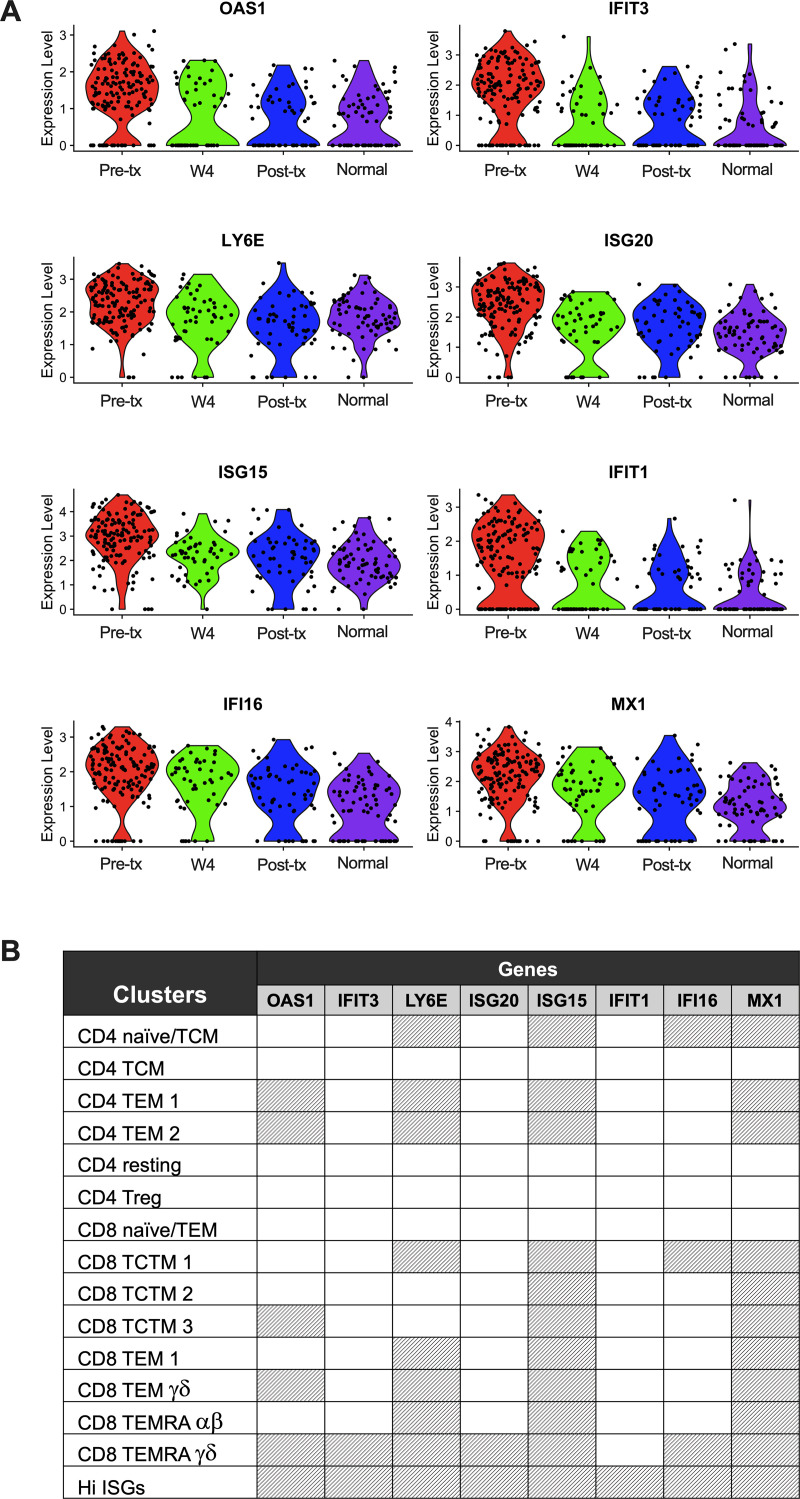
DAA Treatment results in early and sustained reduction in ISG expression in T cells. (**A**) Violin plots of selected Interferon-Stimulated Genes (ISG) that are significantly decreased at the post-treatment timepoint in comparison to pre-treatment samples (adjusted p-value < 0.1). Plot includes only cells from Hi-ISG cluster. The y-axis shows log normalized expression values. (**B**) Table shows the significance of the same genes in each cluster, comparing pre-treatment to post-treatment (grey shaded box = significant). TCM: T central memory; TEM: T effector memory; Treg: T regulatory; TCTM: T central-transitional memory; TEMRA: Terminally Differentiated Effector Memory.

To gain an insight into the kinetics by which DAA mediated HCV cure induces transcriptional changes in T cells in the peripheral blood, we utilized single-cell sequencing to interrogate the transcriptional profile of T cell populations 4 weeks after start of DAA therapy at which point all of the individuals had viral levels <50 IU/mL (**S1 Table**). We found that the decreased innate immune action and IFN-stimulated gene (ISG) expression 12 weeks after completion of DAA therapy was already evident four weeks after the start of DAA therapy (**Fig 4**). When focusing on the ISGs that are differentially regulated in multiple T cell subsets after completion of DAA therapy, we find an early and sustained reduction in the expression of *OAS1*, *IFIT3*, *LY6E*, *ISG20*, *ISG15*, *IFIT1*, *IFI16* and *MX1* (**Fig 4**). While these transcriptional changes were present in a majority of T cell subsets, as shown by the grey shaded boxes in **Fig 4B**, it appears that the high ISG population was the most impacted by DAA therapy, demonstrating significant reduction in these genes to levels found in normal controls.

**Fig 5:**
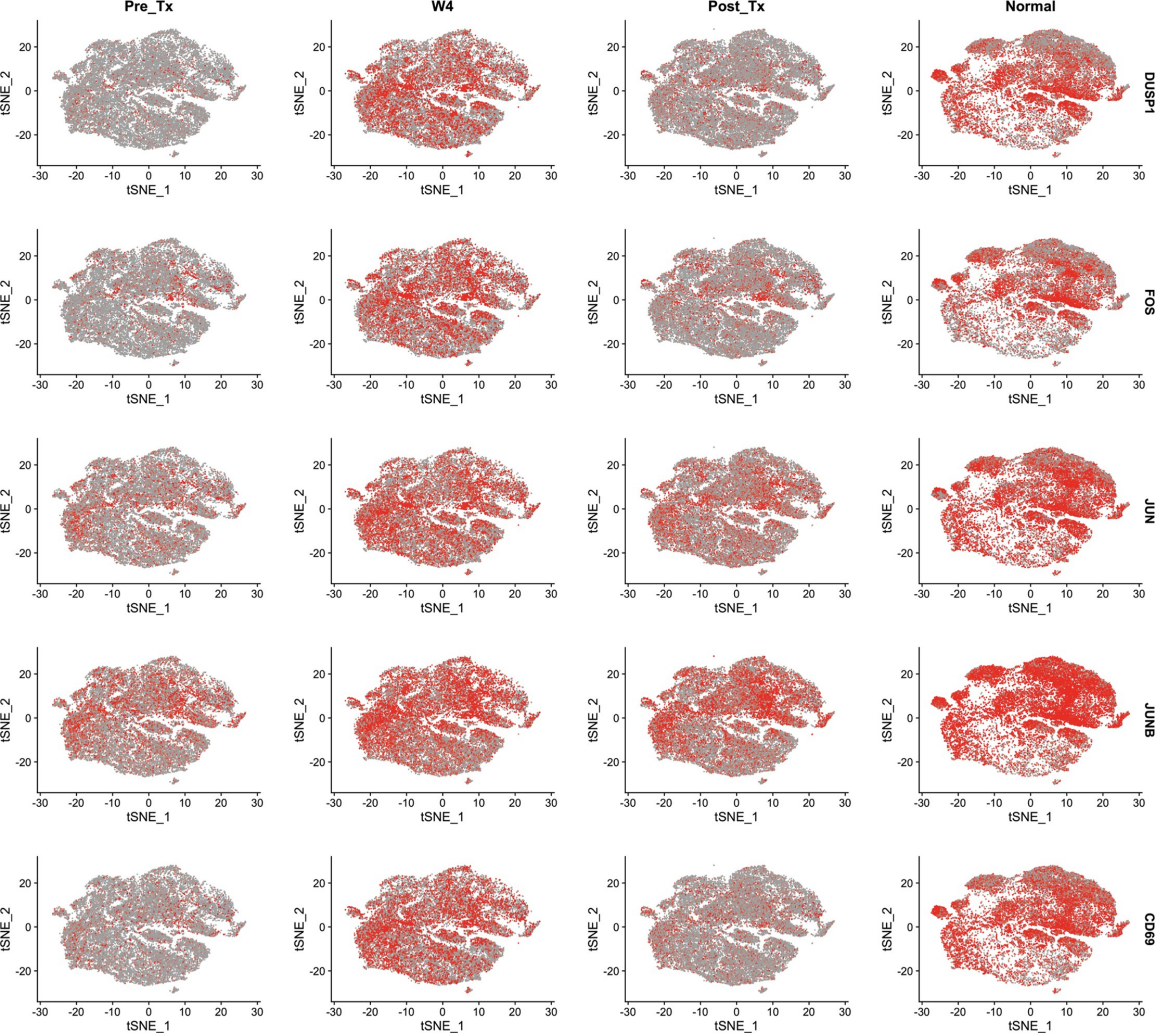
IFN-free DAA therapy results in a transient increase of several transcripts expression associated with T cell activation. Activation markers gene expression projected on tSNE plots. Cells are colored representing the relative gene expression of *DUSP1*, *FOS*, *JUN*, *JUNB* or *CD69* for each of the three time points (pre-treatment, week four of treatment, and post-treatment) and for normal controls. Colors indicate a gradient of gene expression, from red (high expression) to grey (low expression).

Interestingly, the transcriptional changes associated with DAA-mediated rapid viral cure were not exclusively associated with a reduction in IFN signaling. We find a consistent increase in the expression of *DUSP1* and the AP-1 transcription family members *JUN* and *FOS* across all T cell subsets at the week 4 timepoint to levels observed in normal control subjects (**Fig 5**). Furthermore, we observe a transient upregulation of *CD69* expression in the peripherial blood T cells at 4 weeks of DAA therapy which subsequently returns to levels seen pre-treatment. As *DUSP1* is involved in suppressing immune hyperactivation in both T cells and innate immune responses [28–30], these findings suggest that DAA-mediated HCV cure may result in a rapid rebalancing of the T cell compartment in the peripheral blood that results in suppression of IFN-mediated chronic inflammation. However, increased *DUSP1* expression was observed on DAA therapy at the week 4 timepoint, when in most patients viral loads were already below the detection limit and thus, chronic inflammation and ISG expression would be reduced. An alternative explanation could be that activated T cells leave the previously inflamed tissue and enter the circulation upon DAA-mediated resolution of chronic inflammation. However, expression of *DUSP1*, AP-1 family members and *CD69* in normal control subjects is at levels comparable to the week 4 time point suggesting that the week 4 increases in these genes are an indication of transient normalization of the T cell compartment. The expression levels of these genes in control subjects was surprisingly high, therefore we compared expression of these genes to a publically available dataset which used the same chemistry as we had used in our study (https://cf.10xgenomics.com/samples/cell-exp/3.0.0/pbmc_10k_v3/pbmc_10k_v3_filtered_feature_bc_matrix.h5). As shown in **S2 Fig**, there was no significant difference between the levels detected in our control samples compared to the publically available dataset.

## Discussion

Hepatitis C viral RNA is recognized by innate pattern recognition receptors (PRRs) residing within the cytoplasmic or endosomal compartments, including retinoic acid inducible gene-I (RIG-I), Nod-like receptors, and endosomal Toll-like receptor-3 [31]. Activation of PRRs by HCV leads to IFN regulatory factor (IRF)3 phosphorylation, dimerization, and nuclear translocation inducing transcription of type I and III interferon genes. Type I interferons are referred to as “viral” IFNs because they can be induced directly by viral nucleic acids and proteins [32]. Whereas most if not all cells including T cells express the type I IFN receptor chains, T cells do not express IFN-lambda receptor 1 and do not respond to type III IFNs [33–35]. Binding of type I IFNs to their cognate dimeric receptor activates the Janus kinase/signal transducer and activator of transcription (JAK/STAT) pathway, leading to formation of Stat1/2/IRF9 complexes that induce expression of IFN-stimulated genes (ISGs), key players in the innate immune response to viral infection [19]. HCV has developed multiple strategies to establish chronic infection by blocking PRR signaling and the antiviral functions of IFN [36]. In chronic infection, Type I IFNs are key drivers of inflammation and immunosuppression [37]. Hepatic ISG expression has been shown to be a favorable predictor of response to direct-acting antivirals (DAAs), and transcription of ISGs is down-regulated rapidly (within 4 weeks) following initiation of DAAs [4,38–40]. An early study with paired liver biopsies comparing gene expression in hepatic biopsies from patients receiving sofosbuvir plus ribavirin demonstrated that the majority of downregulated genes (65 of 109) were related to endogenous IFN signaling [40]. As most of the liver is composed of parenchymal cells that can respond to both type I and III IFNs, it has been assumed that these effects reflected transcriptional changes within hepatocytes.

Type I IFN can impact T cells at multiple levels, e.g., bolstering cytolytic function in CD8^+^ T cell responses that have antiviral effects early in infection [41] versus promotion of chronic activation and inflammation in later stages that results in progressive immune dysfunction and disease [37]. HCV viral persistence in the face of high ISG expression has been a long-standing paradox. The ability of HCV to establish chronic infection can, in part, be attributed to the several strategies HCV has evolved to antagonize the antiviral actions of ISGs. The emerging concept that IFN-I-driven chronic inflammation and activation in some persistent infections can worsen disease progression has spurred consideration that blocking IFN signaling could reset the immune response [37]. Here, using novel single-cell RNA transcriptomic analyses in patients with chronic HCV infection, we demonstrate that T cells achieve rapid normalization of IFN signaling with DAA therapy. Our data demonstrate that innate immune and IFN-dependent transcriptional responses are rapidly reduced during DAA therapy and maintained well after treatment removal (**Fig 4**). A likely explanation for this is that rapid eradication of HCV results in a dramatic reduction in the levels of systemic IFN; these findings suggest that there is a critical threshold of circulating IFN that is required to maintain the transcriptional changes in T cells associated with chronic HCV infection. Alternatively, these results could suggest a change in the frequency or activation of dendritic cells or other antigen-presenting cells during DAA therapy, a finding that was not directly assessed in our study. Of interest, a recent study has demonstrated that early blockade of type I IFN leads to increased presentation of viral antigens during acute infection leading to better T cell memory [42]. However, it is yet to be determined if rapid normalization of IFN levels in individuals treated with IFN-free DAA therapy impacts T cell function and T cell memory. Although we assessed global T cell responses, the antigen specificities of these T cells, which could have included HCV- or other viral-specific responses, were not assessed; it is also unknown whether antigen-independent mechanisms resulting from changes in the inflammatory milieu could have resulted in these findings [43]. In this regard, we have previously shown that DAAs rapidly downregulate the expression of pro-inflammatory genes in patients and livers of humanized mice [4]. Current technologies do not allow for the analysis of the full repertoire of HCV specific T cells by scRNA-seq. A recent study that pioneered single-cell sequencing of CD8^+^ HCV specific T cells suggests that HCV-specific T cells are contained in several of the clusters defined in our manuscript [44]. Using MHC class II tetramers and conventional RNA-seq, Smits et al. reported that HCV-specific CD4^+^ T cells are dominated by TFH subsets after DAA therapy. Their study found that changes in the frequency of TFH and Th1 subsets was restricted to HCV-specific cells [45]. In our study we did not examine HCV-specific CD4^+^ T cells, however, changes in the frequency of these populations were not observed in the bulk CD4^+^ T cell population.

In addition to the dominant transcriptional changes associated with innate immune activation and IFN-signaling, we identified unique additional transcriptional changes that occur early during DAA therapy. One of these interesting features is the transient increase in expression of *DUSP1*, *JUNB and FOS* in a majority of isolated T cells at treatment week 4. Activation of the AP-1 transcription factor JunB/FOS by different stimuli, such as inflammatory cytokines, stress inducers, or pathogens, promotes both innate and adaptive immunity [46]. AP-1 is believed to directly control the expression of cytokines such as tumor necrosis factor α (TNF-α), and interleukin 1 (IL-1) [47] and plays crucial roles in multiple biological processes in T cells, including the development of T regs and IL-2 signaling [48]. These findings are particularly interesting as *DUSP1* has been shown to negatively regulate pro-inflammatory cytokine production by innate immune cells [49] and is critical for both the activation and proliferation of T cells [28]. Specifically, Zhang *et al* found that anti-influenza T cell responses are impaired in *DUSP1*^*-/-*^ mice [29]. Thus, we hypothesize that the transient activation of *DUSP1* represents a release of the global suppression of T cell responses seen during chronic HCV infection. It is yet unclear if this transcriptional increase in *DUSP1* is due to a decrease in systemic IFN, increased availability of growth factors such as glutocortocoids or cytokines such as IL-7, or related to residual TLR stimulation within these individuals during DAA treatment. Of note, the increase we observe in these genes at week 4 of therapy is comparable to levels detected in normal control subjects and post treatment levels are comparable to pre-treatment levels. This gene expression pattern is consistant with transient release of the HCV-induced transcriptional responses in T cells early in treatment and incomplete restoration of the T cell compartment at the post-treatment time point. The gene expression changes observed in our T cell populations may not be specific to T cells and may also occur in other immune cell populations. In the present study we isolated T cells with greater than 90% purity to generate the most granular T cell single-cell data possible using the 10x platform rather than focusing on other peripheral immune cell populations.

Our studies focused on transcriptional changes in the global T cell compartment as a surrogate for understanding the effects of chronic infection on T cells independent of antigen specificity. As we and others have published that there are global changes in the frequency and phenotype of the global T cell compartment with DAA mediated cure we were surprised to find few consistent changes in the frequency of particular populations of T cells during the course of DAA therapy (**Fig 1**). This discrepancy may be related to the difference between analysis of protein expression via flow cytometry and transcriptional analysis via single-cell sequencing. The single-cell approach does not cluster cells into traditional TH/TFH groups as defined by a small subset of markers but rather on genome-wide gene expression, making direct comparison difficult. We did not observe treatment-specific fluctuations in CD4 subset distributions, however, there are differences when compared to normal control subjects. The genes comprising the CD4 effector mem 1 cluster (TEM1, Fig 1B) suggest an enrichment of Th2 markers (*GATA3*, *TRADD* and *OX40*), as well as markers of T cell activation (*FOS*, *JUN*, *JUNB*). Of interest this population remains significantly higher post-treatment when compared to normal controls. The *KLRB1* associated cluster CD4 effector mem 2 cluster (TEM2, Fig 1B), in addition to *KLRB1*, expresses *TNFRSF25* (Death receptor 3—*DR3*) and *AQP3* (Aquaporin 3), which could indicate this cluster is Th17/TFH-like [50]. However, this population does not show a differential expression of *RORgt*. Our unsupervised clustering did not separate a cluster of cells that express high levels of traditional exhaustion markers (PD1, Tox, TIM-3, etc) as these markers are expressed in many clusters in our dataset. As with the CD4 clusters, changes in the CD8 clusters during therapy are minimal but we do detect a change in the Naïve/TEM CD8 cluster on therapy which decreases at week 4 of therapy and then post-treatment increases compared to week 4, reaching pre-treatment levels but not reaching the levels observed in normal control subjects. None of the other CD8 clusters change with respect to DAA therapy. Most are comparable to normal control subjects with the exception of CD8 TEM1 which remains elevated compared to normal controls. We sampled the peripheral blood compartment because performance of liver biopsy post-DAA treatment is increasingly difficult to justify clinically, considering the high cure rates with standard DAA therapy, potential complications of the procedure, and the non-invasive alternative to assess fibrosis with recent technologies (e.g., fibroscan). It is possible that analyses of intrahepatic T cells during DAA therapy would reveal additional insights. A limitation of our study is that we cannot discriminate antigen specific from non-specific T cells. In addition, it would be valuable to analyze T cells at earlier and at more frequent time points during DAA therapy to understand the dynamics of the T cell response.

In summary, the overall T cell distribution and activation status of bulk T cells after successful treatment of HCV with DAA therapy, when compared to normal control subjects, suggests transient normalization but incomplete restoration of the T cell compartment. In contrast, our results point to a rapid and sustained normalization of IFN signaling with DAA therapy; whether this consistent finding across multiple T cell populations informs other areas such as response to vaccination [11] [3], susceptibility and response to other infections [51], and risk of cancer development post-SVR [52] warrants further study.

## Methods

### Ethics statement

This study was approved by the University of Southern California Institutional Review Board (HS-18-00254). Written and oral consent was obtained from all enrolled subjects.

### Patients

Six male patients with HCV genotype 1 were prospectively enrolled. Blood was collected at baseline, 4 weeks of treatment and 12 weeks after completion of therapy (**S1 Table**). All patients experienced a sustained virologic response. Additionaly, blood samples from three non-HCV subjects were collected to serve as a normal controls.

### Sample preparation

Viably frozen PBMCs were thawed in RPMI containing 10% Human Serum AB (Gemini Bio, West Sacramento, CA). After thawing, cells were washed extensively in Phosphate buffered saline (PBS) and dead cells were removed using an Annexin V dead cell removal kit (Stem Cell technologies, Vancouver, Canada). Following dead cell removal, T cell enrichment was performed using CD3 microbeads according to the manufacturers protocol (Miltenyi Biotech, Bergisch Gladbach, Germany). The enriched T cells (>90% purity by FACs analysis) were then counted, washed with PBS and resuspended at 1000 cells/μl for downstream single-cell capture.

### Single-cell RNA-seq

Single-cell suspensions were processed using Chromium Single Cell 3’ Reagent Kits v3 for 8 of the subjects (including the 3 controls) and v2 for one patient. Briefly, cells resuspended in the reaction mix were loaded into the Chromium Single Cell Chip B, as well as the 10x Barcoded Gel Beads and the partitioning oil. The Chip B was placed into the 10x Chromium controller for cell partitioning, targeting a final number of 10 thousand cells per sample. After partition was concluded, the GEMs (Gel Bead-In EMulsions) were transferred to the C1000 Touch Thermal Cycler for the first phase of reverse transcription and samples proceed to library preparation following manufacturer’s instructions. Libraries were deep sequenced by the USC Molecular Genomics Core, using Illumina NovaSeq6000 platform. The raw data has been deposited in the Gene Expression Omnibus (GEO) repository (NCBI) under the accession number GSE178756.

### Raw data processing

Illumina BCL files were de-multiplexed and converted to FASTQ files using Cell Ranger *mkfastq* (version 5.0.1, 10X Genomics https://support.10xgenomics.com/single-cell-gene-expression/software/downloads/latest). These FASTQ files were then used to quantify gene expression using Cell Ranger *count* (version 5.0.1) and the GRCh38 (version refdata-gex-GRCh38-2020-A, 10X Genomics) human genome reference to generate cell by gene count matrices for each sample.

### Expression data pre-processing

Gene expression counts were processed using the R package *Seurat* (version 4.0.1) [53]. To identify low quality cells and potential cell doublets, we examined the distribution of the number of genes detected in each cell and removed cells with either less than 300 genes detected (low quality) or greater than 3,500 genes (doublets) detected. Cells with greater than 10% of reads mapping to mitochondrial genes were also removed as a high percentage of mitochondrial reads likely represent stressed and/or low-quality cells (**S3 Fig**).

### Sample integration and cluster identification

Samples from all three time points (pre-treatment, 4 weeks, and post-treatment) and normal subjects were integrated into a unified data set using the SCTransform integration workflow implemented in *Seurat* [54]. As part of the workflow, we used reference-based integration [55] by defining the normal control samples as the reference data sets using the *reference* parameter in the *FindIntegrationAnchors* function. Differences in mitochondrial gene content among samples was corrected for during sample integration by passing the percent of reads mapping to mitochondrial genes to the *vars*.*to*.*regress* parameter in the *SCTransform* function. The first 30 PCs were used as the number of dimensions for the *FindNeighbors* and *FindClusters* functions. To find the optimal clustering resolution value we explored a range of resolution values from 0 to 2 by increments of 0.1. We then visualized the range of cluster resolutions by constructing a cluster tree using the R package *clustree* (version 0.4.3) [56]. A final resolution value of 0.8 which identified 23 clusters was selected and used for downstream analyses. Clusters were visualized in two-dimensional space using the *RunTSNE* functions.

### Cell type annotation

We manually curated cell clusters by examining the expression of key marker genes expressed by each cluster. Marker gene expression was visualized among clusters using dot plots and clusters with shared marker gene expression patterns were collapsed. Clusters representing non CD3^+^ T cells (<10% of total individual cells) were removed from the final data set.

### Differential gene expression and pathway analysis

Differentially expressed genes between treatment time points were detected using the *FindMarkers* function in Seurat. An adjusted p-value of 0.1 was used as the significance threshold. Significantly enriched KEGG and Reactome pathways were identified for each cell cluster using Ingenuity Pathway Analysis (IPA, Qiagen).

## Supporting information

S1 Fig Distribution of T cell clusters among patients. tSNE plots of T cells for each patient and each time point. Each row represents an individual patient and columns represent each of the three time points, pre-treatment, week four of treatment, and post-treatment.
(TIF)

S2 Fig Expression of T cell activation marker genes. Violin plots showing the global expression of activation markers in T cell from normal subjects included in this study compared to a publicly available dataset using PBMCs processed with the same 10x genomics kit used for this paper. There were no significant differences between our samples and samples from the pubically available dataset.
(TIF)

S3 Fig Quality control of cells. The relastionship between the number of genes detected and the number of UMIs (unique molecular identifiers) are plotted for each sample after filtering. Each cell is colored by the percent of gene expression from mitochondrial genes.
(TIF)

S1 Table Subject Characteristics. Demographic and relevant clinical data for the subjects included in the study.
(XLSX)
