# Supplementary figures and images for: Single-cell transcriptomic analyses of T cells in chronic HCV-infected patients dominated by DAA-induced interferon signaling changes
Source: PLoS Pathog. 2021 Aug 9;17(8):e1009799. doi: 10.1371/journal.ppat.1009799 (PMC8376199; doi:10.1371/journal.ppat.1009799)

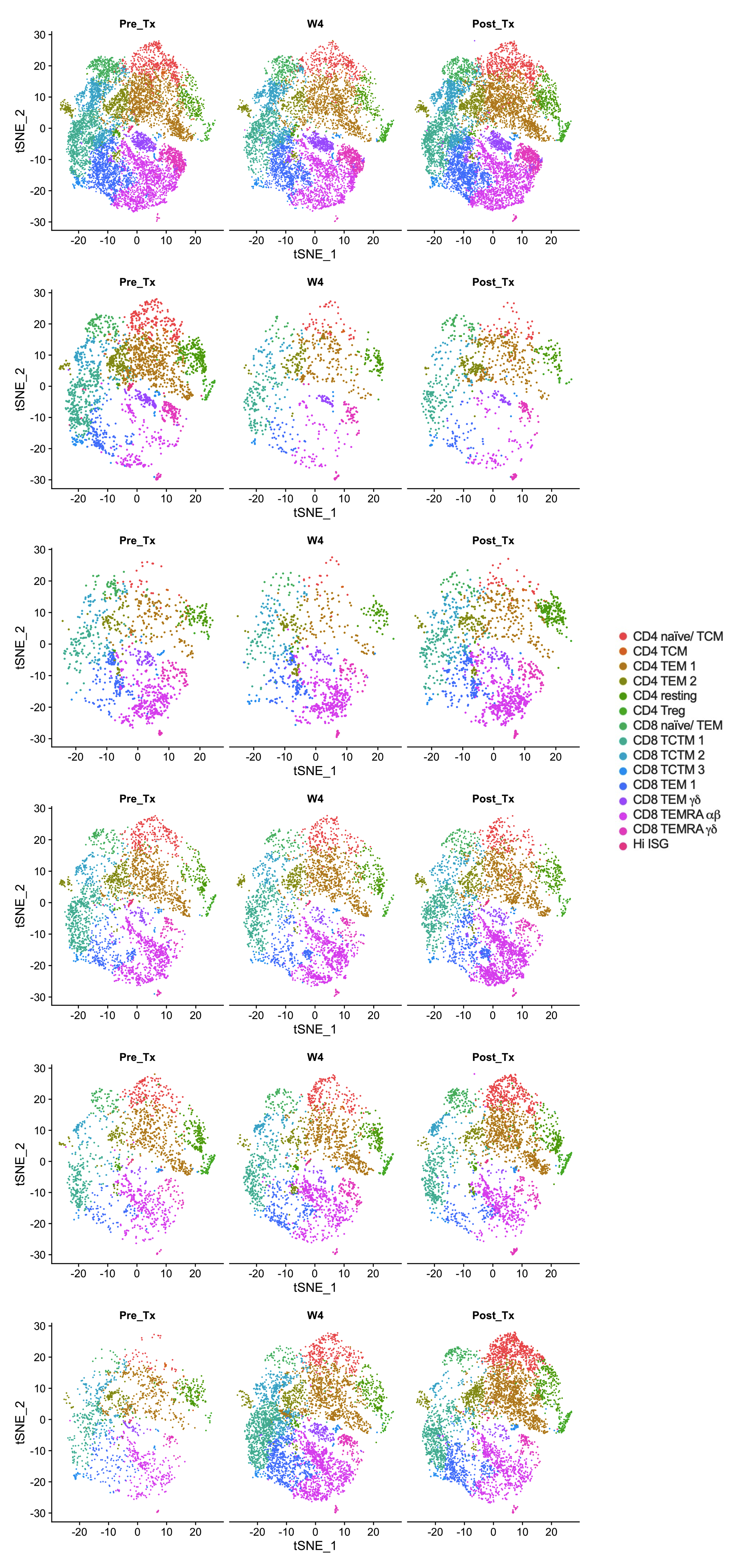

Supplement: S1 Fig — tSNE plots of T cells for each patient and each time point. Each row represents an individual patient and columns represent each of the three time points, pre-treatment, week four of treatment, and post-treatment. (TIF) [file ppat.1009799.s001.tif]

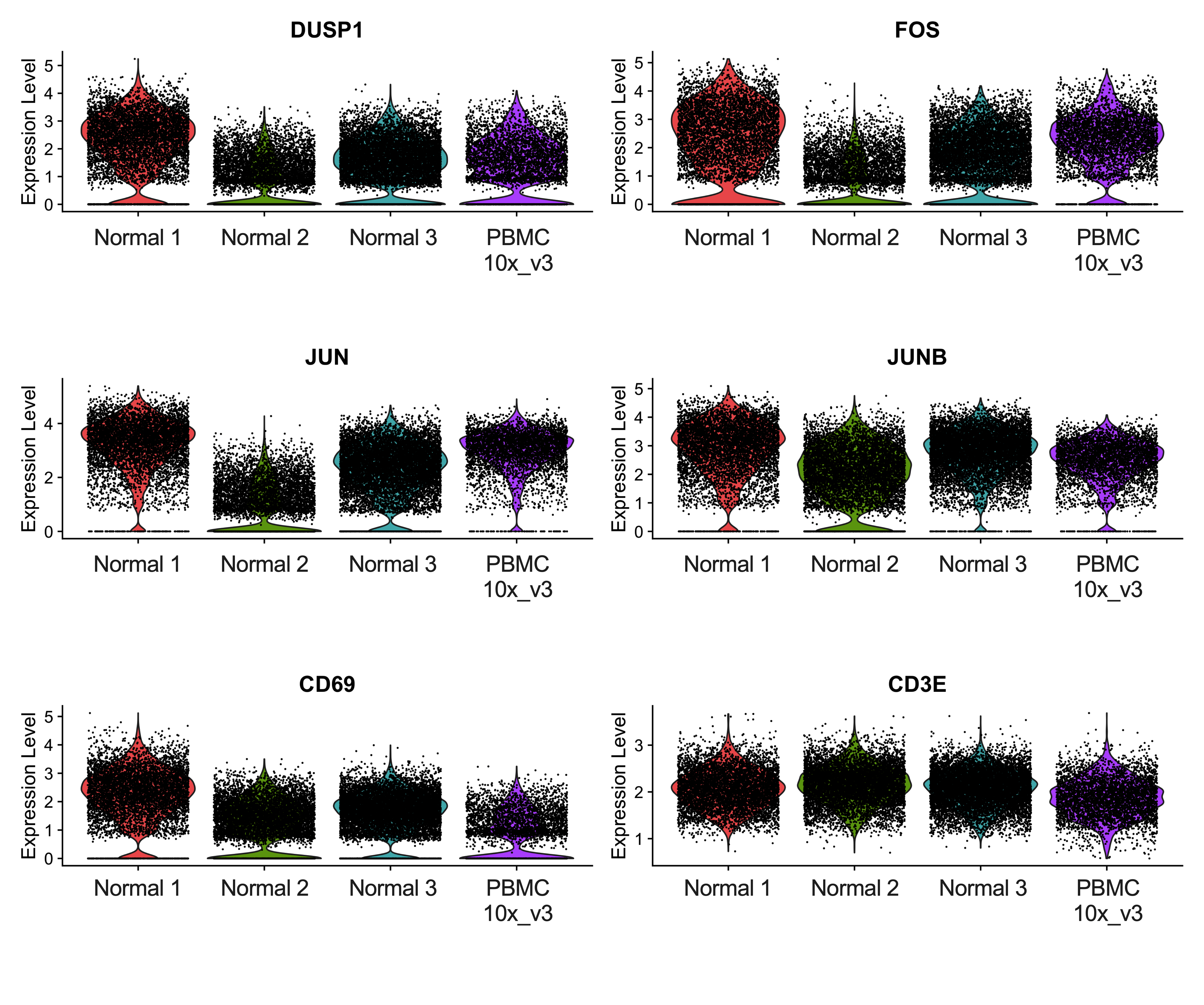

Supplement: S2 Fig — Violin plots showing the global expression of activation markers in T cell from normal subjects included in this study compared to a publicly available dataset using PBMCs processed with the same 10x genomics kit used for this paper. There were no significant differences between our samples and samples from the pubically available dataset. (TIF) [file ppat.1009799.s002.tif]

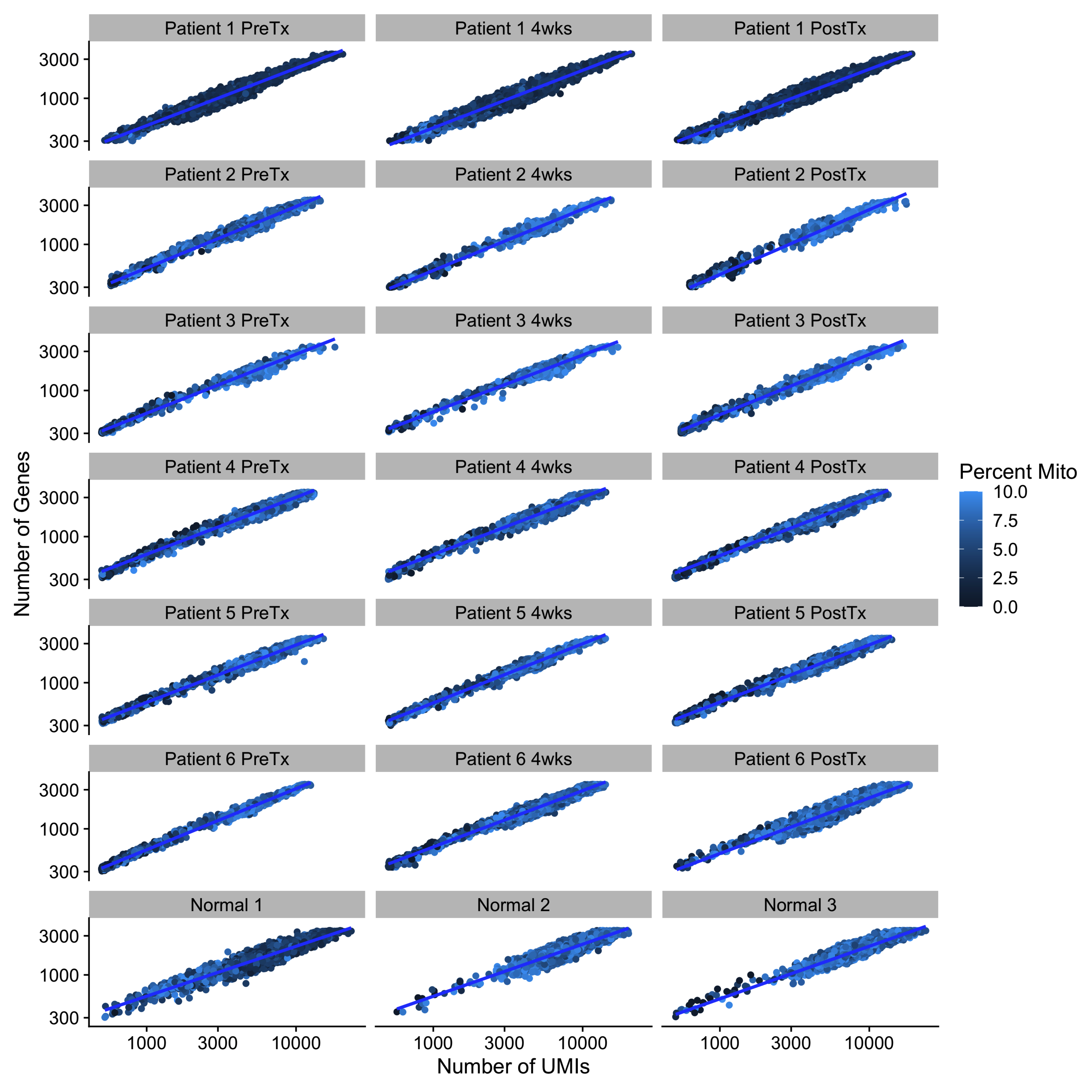

Supplement: S3 Fig — The relastionship between the number of genes detected and the number of UMIs (unique molecular identifiers) are plotted for each sample after filtering. Each cell is colored by the percent of gene expression from mitochondrial genes. (TIF) [file ppat.1009799.s003.tif]
